# Skin emitted volatiles analysis for noninvasive diagnosis: the current advances in sample preparation techniques for biomedical application

**DOI:** 10.1039/d4ra01579g

**Published:** 2024-04-15

**Authors:** Keerthana S., Mohammad Saquib, Harshika Poojary, Gouri Illanad, Divyadarshini Valavan, Selvakumar M, Ramakrishna Nayak, Nirmal Mazumder, Chiranjit Ghosh

**Affiliations:** a Department of Biotechnology, Manipal Institute of Technology, Manipal Academy of Higher Education Manipal Karnataka 576104 India chiranjit.ghosh@manipal.edu; b Department of Chemistry, Manipal Institute of Technology, Manipal Academy of Higher Education Manipal Karnataka 576104 India; c Department of Biotechnology, KLE Technological University Hubballi Karnataka 580021 India; d Department of Humanities and Management, Manipal Institute of Technology, Manipal Academy of Higher Education Manipal Karnataka 576104 India; e Department of Biophysics, Manipal School of Life Sciences, Manipal Academy of Higher Education Manipal Karnataka 576104 India; f Harvard Medical School 25 Shattuck Street Boston 02115 MA USA

## Abstract

Human skin emits a series of volatile compounds from the skin due to various metabolic processes, microbial activity, and several external factors. Changes in the concentration of skin volatile metabolites indicate many diseases, including diabetes, cancer, and infectious diseases. Researchers focused on skin-emitted compounds to gain insight into the pathophysiology of various diseases. In the case of skin volatolomics research, it is noteworthy that sample preparation, sampling protocol, analytical techniques, and comprehensive validation are important for the successful integration of skin metabolic profiles into regular clinical settings. Solid-phase microextraction techniques and polymer-based active sorbent traps were developed to capture the skin-emitted volatile compounds. The primary advantage of these sample preparation techniques is the ability to efficiently and targetedly capture skin metabolites, thus improving the detection of the biomarkers associated with various diseases. In further research, polydimethyl-based patches were utilized for skin research due to their biocompatibility and thermal stability properties. The microextraction sampling tools coupled with high sensitive Gas Chromatography-Mass Spectrometer provided a potential platform for skin volatolomes, thus emerging as a state-of-the-art analytical technique. Later, technological advancements, including the design of wearable sensors, have enriched skin-based research as it can integrate the information from skin-emitted volatile profiles into a portable platform. However, individual-specific hydration, temperature, and skin conditions can influence variations in skin volatile concentration. Considering the subject-specific skin depth, sampling time standardization, and suitable techniques may improve the skin sampling techniques for the potential discovery of various skin-based marker compounds associated with diseases. Here, we have summarised the current research progress, limitations, and technological advances in skin-based sample preparation techniques for disease diagnosis, monitoring, and personalized healthcare applications.

## Abstract 2

Analytical technique for monitoring skin-emitted metabolites to diagnose diseases.

## Graphical abstract

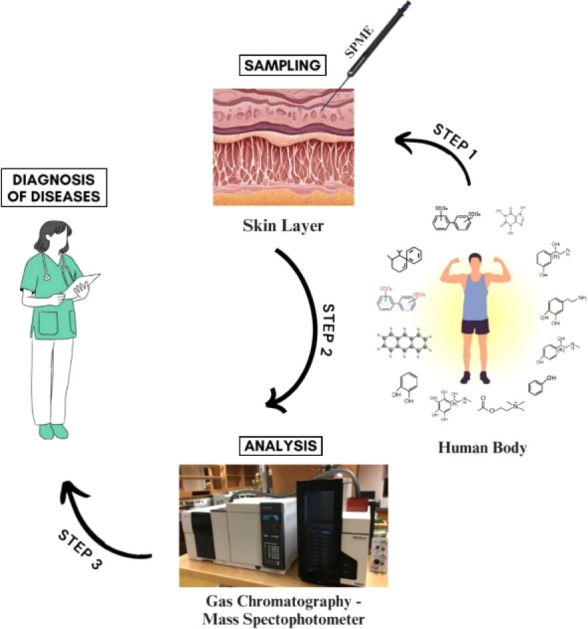

## 1 Introduction

In recent years, skin sampling has gained significant attention in clinical science due to its potential for obtaining valuable physiochemical information. Sweat, produced by eccrine and apocrine glands, and sebum secreted from the sebaceous glands, contains essential components derived from the degradation of proteins and enzymes, enriching the epidermis.^1^ In humans, more than 1800 volatile organic compounds (VOCs) have been identified so far, of which 500 components^2^ are emitted through the skin surface.^3,4^

Initially, researchers focused on collecting sweat from the human body through skin sampling. The primary challenge was collecting an appropriate quantity of sweat to gather biologically relevant information related to the specific diseases.^5^ Researchers have reported using flexible biosensors for sweat sample analysis.^6^ The skin samples were analyzed to determine the metabolites emitted from the human body. Further research has demonstrated the feasibility of using small vessels with organic solvents to transfer the analytes from the skin surface to the analyzer.^7^ However, the practical application of these techniques is limited due to skin irritation and associated discomfort when using non-biocompatible solvents for desorption.

To overcome the limitations of solvent-based extractions of skin-emitted analytes, researchers derived alternative technology where they proposed the use of the Macroduct® sweat collection technique.^8^ Later, simple techniques, like Sarstedt Salivettes cotton swabs and cosmetic pads, were successfully utilized for sweat sampling.^9,10^ These approaches provide more biocompatible and comfortable options for collecting skin samples without the need for solvents. To perform the sample collection from the inner layers of the skin, a solvent-free analytical technique, called solid phase microextraction (SPME) was successfully coupled to the human body for subcutaneous skin sampling.^11^ SPME is a widely used sample preparation tool in analytical chemistry. Developed by Pawliszyn and Arthur, the commercialization of SPME in 1993 increased its utilization and stands out as an innovative approach and user-friendly nature for sample preparation in green analytical chemistry.^12,13^ It is a non-exhaustive technique used for the extraction of analytes from the sample matrix for a specific duration of time. The underlying principle of working of SPME is the occurrence of partitioning equilibria of compounds between sample matrices and adsorbent-coated sorbent materials. The working principle of SPME is as follows:^13^
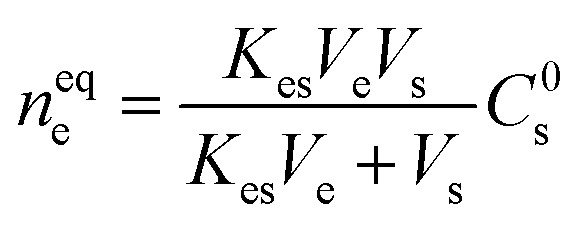
where *n*^eq^_e_ = quantity extracted by the extractant.*V*_e_ = volume of the extractant.*V*_s_ = volume of the sample.*C*^0^_s_ = initial concentration of the analyte until it reaches equilibrium in a given duration.*K*_es_ = partition coefficient or distribution coefficient.

Furthermore, the sorbent trap-based microextraction technique is an active sampling technique.^14^ In this case, the underlying mechanism for trapping the analytes involves diffusion, interception, gravitation and inertial impaction mechanisms, which enable them to immobilize analytes within active sampling sorbent trap.

The application of SPME has been extended to diverse fields, including food quality checks, environmental research,^15,16^ flavor^17^ and fragrance^18^ analysis, clinical chemistry, and the medical field. However, the SPME device is fragile, and therefore, it is sometimes challenging to couple it for the direct collection of metabolites from the skin matrix.^19^

To avoid the complications associated with headspace sampling, researchers developed direct contact sampling from the skin where sorbent materials were embedded or directly placed onto the skin surface for the patch-type sampling.^20^ This sampling system should be elastic enough to conform to the shape of the targeted sampling area, and the materials utilized should be biocompatible to ensure safe and reliable sampling. The technique allows the extraction of volatile and non-volatile metabolites from the skin. However, one of the significant limitations of this technique is the possibility of contamination of the sampling device as the contaminants from the skin or environment may diffuse into the patch, which requires mandatory cleaning of the device before the subsequent sampling. Sometimes, the carry-over from the previous samples may be the source of contamination, particularly in the case of the skin sample analysis. For this purpose, polydimethylsiloxane (PDMS) has gained widespread popularity as the material of choice for skin sampling patches due to its excellent biocompatibility, non-bleeding characteristics during the sampling process, and remarkable thermal and chemical stability.^21,22^ The PDMS membrane, being a liquid material-coated device, allows easy diffusion of metabolites. Later, thin film microextraction (TFME) analytical tools using PDMS membranes were extensively utilized for skin sampling, offering the advantage of efficiency in capturing a large number of metabolites compared to the traditional SPME fiber. PDMS patches have been employed for sampling from various body locations, including the feet, toe clefts, and multiple dorsal and plantar surfaces, to investigate the spatial distribution of bacteria pollution and skin-emitted volatile organic compounds.^23^ The study reported a higher distribution of certain volatiles like butyric, valeric, and acetic acids in the plantar region of the foot compared to the dorsal region. Besides, the influences of body temperature, excretion rates, and skin thickness on skin sampling were highlighted. Importantly, a correlation was observed between human feet emitted skin volatiles and the spatial variations in microbial communities.

To quantify the trace quantity of metabolites, researchers usually coupled the skin sampling devices to highly sensitive equipment like Gas Chromatography-Flame Ionization Detector (GC-FID),^24,25^ Gas Chromatography-Mass Spectrometry (GC-MS),^26,27^ Gas Chromatography Time-of-Flight Mass Spectrometry (GC-TOF-MS) and electronic nose (e-nose).^28,29^ GC-FID is primarily utilized to determine the hydrocarbons and other volatile organic compounds from the skin. Although GC-FID is a potential traditional technique for quantitative metabolite analysis, its limitations lie in monitoring untargeted metabolites. Here, GC-MS provides a potential platform for the identification of untargeted skin volatolome due to the availability of suitable reference libraries and high sensitivity. For quantifications of metabolites, skin researchers utilized GC-TOF-MS to find the biomarkers. However, for rapid olfaction-based research, the electronic nose was utilized for the identification of the limited number of skin metabolites. The advantages of eNose are that it is portable, cost-effective, and does not require highly skilled operators.

This review summarises a comprehensive overview of the recent advancements in sample preparation techniques and their disadvantages for monitoring skin-emitted markers compounds for biomedical research. Additionally, we presented a thorough review of reported metabolites from human skin for early recognition of various diseases.

## 2 Skin sample collection techniques

**Fig. 1:**
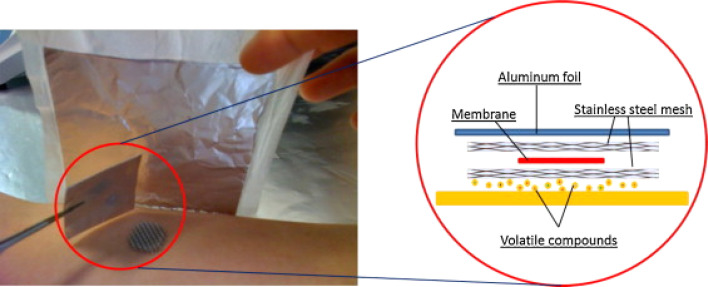
Thin-film microextraction patch for capturing skin-emitted metabolites.

During the last few decades, researchers exploited various sampling techniques to preconcentrate skin metabolites. Sample pretreatment is an important step for the extraction of metabolites.^30–32^ One common technique utilized for this purpose is the solvent extraction technique. Researchers collected the sweat samples by solvent, and the metabolites present in the sample were captured on a cotton pad for further extraction with suitable solvents like hexane, dichloromethane, or ether.^33,34^ Subsequently, solid-phase micro-extraction (SPME) was reported as an essential technique for preconcentrating volatile metabolites. The solvent-free SPME technique was utilized for the metabolite extraction from the saliva and skin matrixes.^35^ Duffy *et al.* described a wearable headspace solid-phase microextraction technique designed to monitor the natural baseline skin volatiles and their subsequent modifications after applying a fragrance to the skin surface.^36^ They utilized gas chromatography-mass spectrometry to analyze the scent profile, but they needed more attention in extracting hydrophilic compounds. The application of SPME has expanded beyond its initial purpose of headspace extraction. The SPME probes were reported for utilization in the tissue sampling of living organisms (*in vivo* SPME), enabling the capture of active components.^36,37^ In a further study, researchers used glass beads as the active sorbent materials, and the beads were rubbed on the hands or feet to extract the metabolites.^38^ Subsequently, body odour was collected from different body parts, including the hand, where fewer sebaceous glands, and apocrine glands are absent.^39^ The study was successful in determining the association between the physical character of the human body and the skin-emitted gases. Fig. 1 depicts the procedure of skin sampling using an SPME-based patch on the skin.^40^

Apart from SPME, investigators utilized a dynamic headspace adsorption technique with various active polymeric sorbent materials to extract metabolites from the skin surface. This approach typically involves absorbing skin compounds onto gauze, cotton pads, or worn clothing. The preconcentrated compounds were released from the substrates and reconcentrated onto adsorbent traps (glass tubes filled with Porapak or Tenax absorbent).^41,42^ However, the use of the support medium, like gauze and cotton, for skin sampling provides some disadvantages for the quantification of trace levels of skin-emitted compounds. Also, certain compounds emitted from the skin cannot effectively transfer to the sample matrix through diffusion. Furthermore, despite undergoing sterilization processes like autoclaving and gamma radiation, analysis of the headspace of sterile gauze pads revealed the presence of various volatile organic compounds. Therefore, the collection medium may limit the accuracy of metabolic profiling in skin research.

To overcome the issue of the fragile nature of SPME and by enhancing the adsorption efficiency of trace quantity of analytes, a versatile and effective technique to collect small metabolites produced from the skin is the “sorptive tape” extraction method, which is analogous to the thin film microextraction technique.^43^ With this approach, *in vivo* sampling was possible within a certain time, and this also reduced the environmental contamination. The sorptive tape may be connected directly to a GC device for thermal desorption. This technique has been used to analyze the skin-emitted volatile metabolites. To ensure repeatability, minimize sample loss and external contamination, Pawliszyn and colleagues reported a study where the thin PDMS sorptive pad captured the skin VOCs from the human body.^40^

In another study, volatile emission tobacco smoke, the MonoTrap DCC18 sampling device was successfully connected to the skin using a passive sampling flux technique.^44^ However, a significant limitation of this method was the requirement for prior knowledge of the flux flow to operate it effectively. Kimura *et al.* introduced the novel noninvasive passive flux sampler (PFS) to estimate age-related VOCs from the skin.^45^ The study reported the enrichment of 2-nonenal with age and the flux of diacetyl, which was at its maximum at 30 years old but decreased after 40 years. Nevertheless, quantifying VOCs from the skin using this device required a cumbersome sampling method. In another study by Zeng Qingya *et al.* reported exciting outcomes after introducing a minimally invasive technique that utilized immunodiagnostic microneedles to extract volatile organic compounds (VOCs) from the skin.^46^ They successfully demonstrated that polyacetic acid-coated microneedles could extract analytes from the skin of mice, and the results were comparable to the conventional immunoassays. This research was performed directly on animal model for the extraction of skin metabolites. However, further research is necessary to optimize the coating of the microneedles for effective sample preparation.

In a further study,^39^ researchers provided each participant with a polyvinyl fluoride resin film bag filled with nitrogen gas to collect skin volatiles. After the collection period, gas samples were transferred to a storage bag attached to a stopcock, which served as a sample reservoir for the research. The study demonstrated a promising approach for skin research. In the previous study on breast cancer, investigators collected sweat samples from the breast using a kit that included a sterile cotton pad, jar, soap, cotton tissues, and compression bandage. Here, they used four SorbStars® polymers, which were rubbed onto the individuals' hands for 15 minutes to absorb hand perspiration to prevent contamination^47^ DeGreeff *et al.* performed a study where the volatile compounds were collected using non-contact, airflow-based sampling, and SPME-GC/MS techniques, employing sorbent materials.^48^ Their research investigated the odour collection and analysis using a dynamic real-time headspace concentration device with the pre-determined sampling rate, suitable sorbent materials, and some accessories (polyester material, cotton-blend gauze, *etc*). Their research exhibited a promising outcome in the skin sampling approach.

Skin treatment procedures prior to odour collection vary significantly across different studies. In many cases, volunteers were instructed to adhere to the specific guidelines regarding their diet and the use of scented soap/shampoo on their skin before the experiments. Researchers analyzed human skin emissions and identified the volatile molecules that might act as attractants for the Yellow Fever mosquito species.^49,50^ Gallagher *et al.* limited the use of underarm deodorants/antiperspirants, colognes, or sprays during the experiments.^7^ Polydimethylsiloxane passive samplers were utilized to capture skin-emitted volatiles from ankle and wrist surface regions.^51^ In addition, investigators utilized wearable colourimetric sensors to monitor skin-emitted ammonia levels in individuals to determine surface pH.^52^ Toma *et al.* performed a study using a customized system to monitor ethanol vapour from the ear. They utilized an ethanol bio-sniffer coupled to an over-ear collection cell for real-time measurement of skin-emitted ethanol vapour after alcohol intake, suggesting a potential method for monitoring blood ethanol in a noninvasive way.^53,54^ An attempt was also made by the researchers to understand age-related disorders through quantifying *trans*-2-nonenal vapor with the help of liquid- and gas-phase biosensors (bio-sniffers). However, the application of the sensors was limited to the effective determination of the dynamics of skin gases in real-time. Therefore, the study was not clinically viable for the practical application of the reported sensors in the absence of potential sensitivity.^55^

Apart from the microextraction techniques, several biosensors have been reported for medical applications, including sweat-based biosensors for blood glucose monitoring purposes. Due to the excellent optical properties, nanomaterials-based surface-enhanced Raman scattering biosensors were reported for monitoring metabolites.^56^ Recent advancements led the researchers to develop an innovative device that uses skin sensors to track lifestyle habits.^57^ By analyzing volatile organic compounds present in the skin, investigators were able to distinguish among fasting, non-fasting, and alcohol states.^58^ The device was equipped with standard components, and it was connected *via* Wi-Fi and Bluetooth. Thus, it was able to provide real-time insights into an individual's lifestyle, which might be valuable information for clinical applications. Similarly, a wearable sensor was designed to measure lactate concentration in sweat. These wearable electronics were cheap, wireless, and easy to use. The proposed device was independent of the sweat flow rate from the human body. Therefore, it can be utilized for hypoxia-related therapy.^59^

**Fig. 2:**
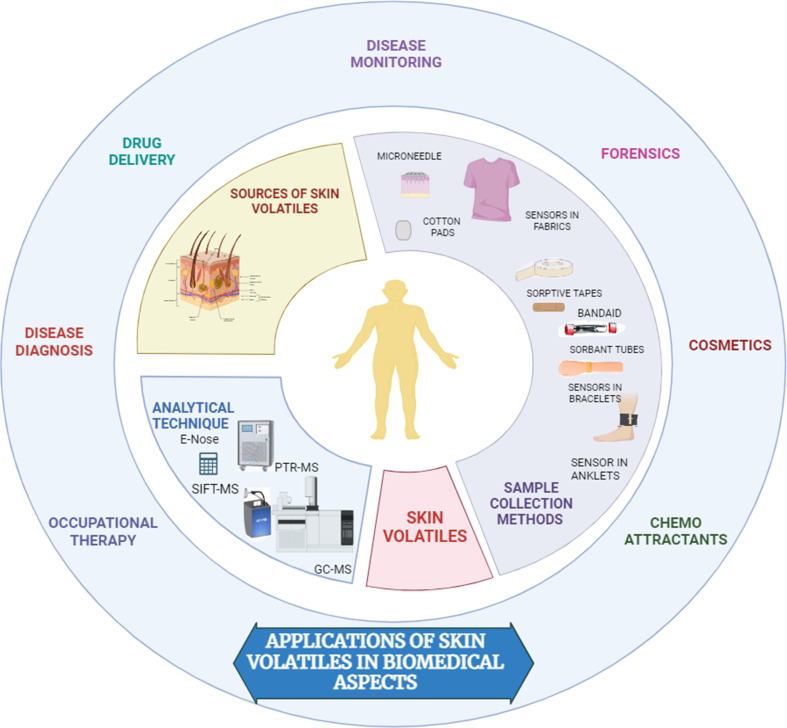
Pictorial representation of human skin sampling techniques.

The application of a simple wearable platform incorporating pH-responsive sensor spots for the measurement of skin surface pH *via* the volatile ammonia emission from the skin in a healthy participant study was reported. Fig. 2 demonstrates the various skin sampling techniques for biomedical applications.

Finally, the traditional analytical techniques, including skin bags, swab collection, and tape stripping methods have some advantages and disadvantages for the skin volatile study. Although a few of these techniques are easy-to-perform, the extraction efficiency of some techniques for skin metabolites is limited^60^ Therefore, it is very important to choose a suitable technique for the analysis of human skin-emitted volatiles.

## 3 Skin-emitted volatolomes and their association with diseases

Researchers reported various marker compounds in skin samples, and this information has been utilized to interpret the physiological profile of the subjects.^4^ It is important to note that sampling procedures and target areas for skin sampling should be considered to interpret the concentration of marker compounds associated with various diseases. In the last few years, hundreds of skin-emitted metabolites have been reported in skin samples, and several studies have utilized the metabolic profile for early diagnosis of diseases.

Syed *et al.* conducted an olfactory-based study to monitor the semiochemicals from humans and birds.^61^ They summarised a list of compounds (6-methyl-5-hepten-2-one, nonanal, decanal, and geranylacetone) emitted by humans and birds that serve as attractants for *Culex* mosquitoes. While nonanal compounds played a potential role in bird odour profiles for attracting specific mosquito species. However, the study did not mention the possible variation in the olfactory profile of individuals from various continents of the population and the various species of mosquitoes.

Dustin J Penn *et al.* conducted a study directly on a large number of individuals from an Austrian Alps village to collect the axillary sweat, urine, and saliva and finally derive the gender-specific metabolic fingerprints within the population.^62^ The investigators identified twelve compounds (ketone, 6-phenylundecane, pentadecanoic acid, hexadecanoic acid, a methylhexadecanoic acid, heptadecanoic acid, a dialkyl ether, nonadecane, isopropyl hexadecanoate, 2-ethyl-hexyl-4-methoxycinnamate, docosane, 1-octyl-4-methoxycinnamate) which can help to characterize the gender within the population. However, the study found no unique skin-volatile for distinguishing the sexes. Instead, it was possible to distinguish the gender by multivariate distribution of datasets. This study demonstrated the sex-specific odours for discriminating the subjects and sexes. However, more research is required to implement the study outcome to a potential platform (like e-nose) for biometric fingerprinting and screening of diseases.

To get insight into specific sweat metabolites associated with Behcet's disease (BD), an autoimmune disease, sweat samples were collected from 38 volunteers.^63^ After analysis of the sweat metabolite profile, researchers observed the association of a few skin volatiles including citrulline, pyroglutamic acid, urocanic acid, 2-oxoadipic acid, cholesterol 3-sulfate and pentadecanoic acid with BD diseases. This study unveiled the role of 2-oxoadipic acid in sweat samples in autoimmune disease. As the mentioned compound is the primary catabolic metabolite of tryptophan, this study indicates the pathways for triggering the said autoimmune disease. Therefore, quantification of low levels of 2-oxo adipic acid metabolites may indicate the progression of BD disease. However, the study is limited for clinical application due to the small sample size and inadequate accuracy for discriminating active and inactive BD.

**Fig. 3:**
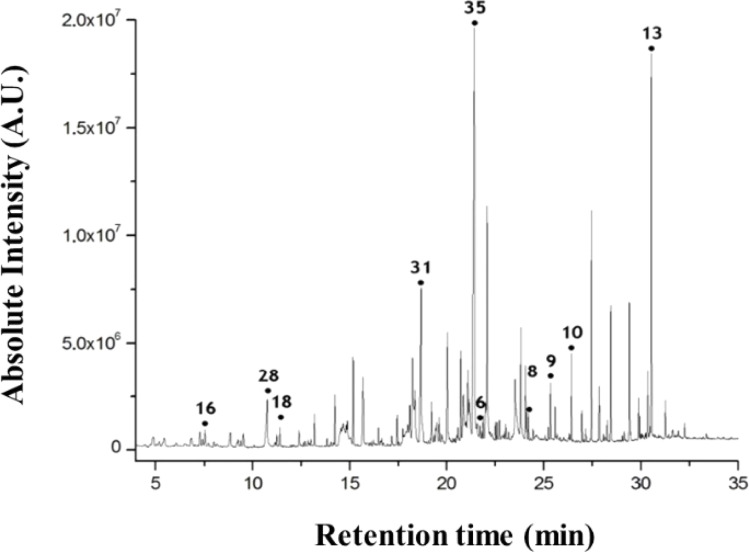
Human scent chromatogram by headspace-gas chromatography-mass spectrometry analysis.

Furthermore, skin research has extended to early diagnosis of pre-menstrual syndrome.^64^ Researchers reported skin volatiles during the menstrual cycle in women. To study the variation of skin metabolic profile during the menstrual cycle, researchers collected the skin-emitted gas samples and investigated the influence of the emission flux on the skin gas sample during the four phases of the menstrual cycle. In the study, the authors considered about 65 volatile compounds after analysis of skin volatiles data. According to the study, 3-hydroxy-3-methylhexanoic acid in the axial sweat and 3-methyl-2-hexenoic acid present in the axial odour were observed to be associated with the menstrual cycle. The researchers observed that during the menstrual M phase, the levels of 1-hexanol and propanal compounds were increased compared to the other phases, while volatiles such as ethyl mercaptan and butyric acid were present in low quantities within the skin samples. Furthermore, investigators utilized the human skin volatiles to check the feasibility for distinguishing the male and females. From this study, researchers reported some volatile compounds (Fig. 3) in the human scent chromatogram including nonanal (16), nonanoic acid (28), undecanal(18), tetradecanoic acid(31), hexadecanoic acid(35), eicosane(6), docosane(8), tricosane(9), tetracosane(10), and squalene(13).^65^

Researchers utilized the PDMS-coated patch, which can be employed for direct sampling by attaching it directly to the skin surface, where the PDMS material is positioned at a specific distance from the skin surface to capture volatile analytes. Headspace sampling effectively eliminates potential matrix contamination from substances like dust particles and other chemicals present on the skin. However, a study by Jiang *et al.* comparing the extraction of volatile organic compounds (VOCs) between these methods found no significant difference, except for the direct coupling method, which yielded higher intensities for semi- and low-volatile compounds like 1-tetradecanol and 1-octadecanol.^22^ Direct contact skin sampling is particularly advantageous when evaluating the total number of metabolites emitting from the skin surface. Consequently, various clinical applications have increasingly adopted direct sampling through the PDMS membrane. Direct contact with VOCs, particularly skin contact, as well as inhalation, can have a negative impact on one's health. Skin irritation may arise from exposure to limonene and diethyl phthalate, whereas 2-propanol can cause allergic responses in certain people.^66^

Research conducted by Schivo *et al.* utilized an animal model to examine the histological evidence of early-stage ulcer development after sampling from an ear attached to PDMS patches.^67^ The study focused on fifteen skin-related compounds, and the twelve compounds demonstrated differentiation between healthy and ulcer groups. Martin *et al.* conducted a study investigating the correlation between skin metabolites and body odour, focusing on the impact of a single nucleotide polymorphism (538G A in the ABCC11 gene) on the concentrations of apocrine-derived axillary odour molecules. They identified four volatile fatty acid compounds for this purpose.^68^ This study may be helpful for application in clinical metabolomics, chemical ecology, and forensic research.

To profile the whole-body volatiles, researchers collected headspace samples from the human body in a customized and isolated chamber. They reported 43 VOCs (nonanal, benzyl alcohol, linalool, benzaldehyde, ethylbenzene, *etc*) in the headspace of the body, including acetoin, long-chain aldehydes, and squalene. Aldehydes, including hexanal, heptanal, octanal, nonanal, and decanal were enriched in the sample matrix.

Researchers performed the sweat test to check the feasibility of diagnosing cystic fibrosis.^69^ The study may be utilized in clinical settings where test accuracy is less important for the diagnosis of the disease. Sweat discharged from the skin's surface was found to include several pharmacological metabolites, including tetrahydrocannabinol, opioids, and 3,4-methylenedioxymethamphetamine. The development of new drugs for treating skin conditions or cosmetics may be facilitated by understanding how the skin can metabolize therapeutic substances, consumer goods, or cosmetics.^70^ Various metabolites identified from different parts of the body have been listed in Table 1.

**Table 1:** List of various skin sampling techniques and reported marker compounds for biomedical applications

Sampling method	Analytical technique	Materials used	Sample size	Analytes reported	References
Active sampling by sorbent traps	GC-MS and HPLC	3 Tenax tubes and 1 DNPH	Single subject	37 VOCs reported (predominant compounds are 6-methyl-5-hepten-2-one, nonanal, decanal and 6,10-dimethyl-5,9-decadien-2-one)	71
Passive sampling by patch	GC-MS and nanomaterial-based sensors	PDMS pouches and PDMS sheets	636 subjects aged 22–60 years	Toluene	72
Passive sampling by patch	HS-GC-MS	Pharm Chek® patches (adsorbent pad coated with polyurethane film with hypoallergic glue)	32 control subjects and 32 cancer patients (adenocarcinoma)	Total n-aldehydes presented to be strongly correlated with staging of adenocarcinomas, while phenol and 2,6-dimethyl-7-octen-2-ol were correlated with Gleason score in pancreatic cancer	73
Passive sampling	GC-MS	Polyvinyl fluoride resin film (Tedlar®)	40 Japanese women aged 35–44 years	Allyl mercaptan and dimethyl trisulfide	74
Active sampling by sorbent traps	GC-MS	Four sets of Tenax-TA tube and three sets of DNPH with ozone scrubber	14 young adults (7 women and 7 men)	Ketones, siloxanes, aldehydes, alkenes and alcohols and 42 VOCs were compared	75
Passive sampling and active sampling	GC × GC-HRMS, GC × GC-Q-TOF MS	Dermatess sweat pads made up of viscose and polyester, Tenax-GR (poly-2,6-diphenylphenylene oxide with 23% carbon, 60/80 mesh, average particle size 0.5 μm)	40 subjects (18 women and 22 men −80 samples)	326 compounds from a diverse range of chemical classes (278 identified compounds, 39 class unknowns, and 9 true unknowns)	76
Active and passive sampling	DHS-TD-GC-MS	Gauze swabs and Tenax TA adsorbent tube	64 participants (21 controls and 43 Parkinson's disease subjects)	Perillic aldehyde, hippuric acid, octadecanal and eicosane	77
Passive sampling	GC-MS and LC-MS/MS	Gauzes, Diflan or ashless filter papers and glass Pasteur pipette with a latex bulb (supplied by Scharlab)	6 healthy volunteers (3 women and 3 men)	Benzenoids, carbohydrates, amino acids, carbolic acids and derivates, flavonoid, lipids, 1-monolaurylglycerol, 1-monomyristylglycerol, nucleosides, analogues, organic nitrogen, oxygen, phoshphoric acid compounds, organoheterocylic compounds and oxoanionic compounds	78
Artificial olfactory system	E-nose	The sensors used are of the TGS 822, TGS 2612, TGS 2620, TGS 826, TGS 2603, TGS 2600, and TGS 813 types	17 female and 7 males over age 19 years	trimethylamine-*N*-oxide (TMAO)	79
Active sampling	GC-IMS	Tenax adsorption tubes	7 healthy volunteers (4 women and 3 men) between 27-50 years of age	27 compounds were identified, 14 of them could be clearly assigned to sweat (1-decanol, 2-decanol, acetophenone, 6-methyl-5-heptene-2-one, 5-methyl-heptane-3-one, 2-ethyl-1-hexanol, propionaldehyde, 2-hexanone, 1-pentanol, 2-hexanol, 1-nonanol, pelargonaldehyde, propane acid	80
Passive sampling	HS-GC/MS	Tight t-shirts made up of 50% cotton and 50% polyester	50 healthy volunteers (27 women and 23 men)	6-Methyl-5-hepten-2-one	81
Active sampling	GC-ICP-MS	Dual sorbent tube containing Tenax TA and Carbograph 5TD (Markes international Ltd.)	An adult male cadaver aged 74, weighing between 60 and 65 kg at the time of death	37 compounds-aldehydes (hexanal, nonanal, decanal), acids (nonanoic, decanoic, dodecanoic, tetradecanoic and pentadecanoic acids) and hydrocarbons (squalane, squalene)	82
Passive sampling	GC-FID GC-MS and GC-GC × MS (Marker peak identification and comparison)	Two cotton rods (0.8 × 2.5 cm)	368 participants, 82 COVID19 positive patients and 268 COVID19 negative volunteers	*p*-cymene	83
Passive sampling	HS SPME GC × GC-MS	Two cotton rods (0.8 × 2.5 cm)	66 subjects were analyzed. 30 COVID-19-positive patients (12 proven by RT-PCR and 18 proven by antigen test kits, Singclean (Nasal swab), China) and 36 negative volunteers (14 and 22 proven by RT-PCR and the antigen test kits, respectively)	Total of 233 volatile metabolites. After using feature selection by FS-CR, 14 significant metabolites were revealed which included *p*-cymene, linalool and 2,6,11-trimethyldodecane	84
Commercial AbsoluteIDQ p180 kit	QTRAP 4500 MS system and absolute IDQ p180 kit	NA	15 patients with Atopic dermatitis (11 women, 4 men, ages 20–50 years) and 17 controls (7 women, 10 men, ages 23–75 years)	Total of 77 metabolites were found – Amino acids, biogenic amines, acylcarnities, sphingomyelins, glycerophospholipids	85
Passive sampling	Differential Chemical Isotope Labeling (CIL) LC-MS	Patch consisting of a Tegaderm film and two layers of Whatman filter paper, gauze sponge sweat patch	20 subjects (10 male and 10 female)	3140 sweat metabolites, 84 identified, and 2716 mass-matched to metabolome databases	86
Commercial Absolute IDQ p180 kit	“Nano-DESI mass-spectrometry and AbsoluteIDQ p180	Polytetrafluoroethylene (All-Fluoro), acrylonitrile butadiene styrene polymer probe made up 5 micropatches attached with the adhesive bandage tape	100 psoriatic patients and 100 control group	Several polar metabolites, mainly choline and glutamic acid	87
Passive sampling	GC × GC-TOF-MS	Passive PDMS samplers designed as loop with uncoated silica capillary column	20 subjects aged 20–59 years	5-Ethyl-1,2,3,4-tetrahydronaphthalene, 1,1′-oxybisoctane, 2-(dodecyloxy) ethanol, α,α-dimethylbenzene methanol, methyl salicylate, 2,6,10,14-tetramethylhexadecane, 1,2-benzenedicarboxylic acid, bis(2-methylpropyl) ester, 4-methylbenzaldehyde, 2,6-diisopropylnaphthalene, *n*-hexadecanoic acid, and *γ*-oxobenzenebutanoic acid ethyl ester	51

In another investigation, researchers aimed to identify stress-related biomarkers after placing PDMS membranes on subjects' foreheads and extracting compounds from the skin surface.^88^ They observed that compounds such as 3-carene, benzoic acid, and *N*-decanoic acid were associated with the metabolic response to stress.^89^

The skin is known to be exposed to moderate concentrations of volatile compounds through the surrounding air and low concentrations of volatile compounds in the liquid phase. The rate and amount of absorption are significantly influenced by experimental conditions, solvent properties, and individual variations among study participants. Factors like hydration, temperature, skin conditions, *etc.*, affect the absorption. The outermost layer (stratum corneum) of the epidermis is responsible for impaired permeability of the skin as it prevents the diffusion of volatile organic compounds originating from the hypodermis, dermis, and the other layers of the epidermis. To overcome this obstacle, researchers have developed various techniques, including iontophoresis,^90,91^ electroporation^92–94^ photomechanical waves,^95,96^ and microneedle array.^92,97^

The stratum corneum is composed of lipid and polar regions and preferentially absorbs lipid soluble and polar compounds.^98^ Studies have shown that compounds such as tetrachloride and 1,1,1-trichloroethane, which are water-soluble, penetrate the skin better when the skin suffers from water loss.^99,100^ On the other hand, skin hydration significantly increases the absorption of water-soluble compounds.^101,102^ It was also noted that the absence or presence of inferior stratum corneum would increase *trans*-epidermal water loss, thus providing little to no resistance against penetration of VOCs.^103^ Percutaneous absorption is known to be enhanced by repeated sweating. It was observed that pure liquid VOCs showed less permeability due to the dehydration of the stratum corneum.^104,105^ Increased skin temperature enhances percutaneous absorption proportionally. In most studies, skin absorption measurements were conducted in temperature-controlled experiments.

## 4 Current challenges of skin sampling

Although human skin sampling provides a potential platform for noninvasive disease screening, there are several challenges, including the variability among subjects, individuals' skin depth, and chances of contamination during sampling. The major limiting factor for skin sampling is the possibility of contamination from exogenous sources, including the use of perfumes, environmental exposure during sampling, and food consumption by the participants.^4^ The change in skin thickness may impact the diffusion of marker molecules through the skin surface. Therefore, it may alter the skin metabolite concentration due to variations in skin depth. Additionally, the choice of the sampling area and timing can significantly impact the measured data due to variations in diffusion rates across different skin regions.^40^ The sampling time should be appropriately considered to ensure the repeatability of the data. The individual's skin is associated with the variation of the lipid composition and moisture content, suggesting the possible interpretation of the skin-emitted volatile composition during skin sampling within the community. To overcome those challenges, skin sampling requires the standardization of the sampling protocol and the use of sensitive analytical tools for capturing the volatile metabolites. Individual's physical parameters may be considered to report the concentration of metabolites emitted from the skin matrix.^106^ Also, the *trans*-epidermal water loss could be used to correct the profiling of skin metabolites. Interpersonal variation, low analyte concentrations, the complexity of calculating absolute concentrations across time, and the small number of biological specimens collected are difficulties for skin metabolomics. Since the skin is a dynamic matrix, metabolite levels alter throughout time. The analytical process, including sampling and detection, further influences variability. Passive sweat sample collection by microfluidic technology has several advantages, including fast measurement, easy handling, biocompatibility, and cost-effectiveness to traditional techniques. However, this technique is also limited due to unreliable results, lack of accuracy, contamination, and evaporation of sweat from human skin samples.^107,108^

## 5 Summary

Skin volatile research has emerged as a potential topic for application in healthcare, diagnostics, and forensic studies. The human blood consists of hundreds of metabolites, sometimes produced by certain microbes associated with various diseases. Apart from the metabolic process, the volatile compounds may be produced from environmental contamination and the activities of the pathogens on the skin. Researchers tried to gain insight into the disease states of persons and the follow-up after the therapy for a better understanding of the health feedback. The identification of multiple volatiles associated with various diseases may facilitate the design of healthcare devices in the future. It is possible to interpret the physiological pathways associated with various conditions in the human body. Nowadays, skin research has gained interest in the beauty and fragrance industry, where commercial companies can customize their cosmetics products based on the subject's unique scent profile. Each person has a unique scent profile, which can be utilized for forensic studies. However, technological development is required to produce analytical products as an outcome of skin research with substantial commercial interest. Although GC-MS and electronic noses show the potential technique for human odour profiling, more research is needed to fully understand the pathophysiology associated with various human conditions and design reliable products. Although current advances in sample preparation techniques for biomedical applications, especially skin volatiles analysis, are notably significant, future studies are required for the successful implication of skin-based research outcomes in regular clinical settings. It is important to design automatic and high-throughput techniques for the analysis of a large number of skin samples within a certain time. More research is required to miniaturize the sampling tool so that it can effectively extract a large number of compounds in a short time frame. Human skin emits oil, and this may saturate the passive sampling device (like a patch) after a certain time for skin volatiles extraction. Therefore, the researchers may consider this fact and design the sampling tool to increase the sensitivity of the technique. To facilitate skin VOCs-based research for clinical application, researchers may incorporate artificial intelligence (AI) and machine learning processes to analyze the data, identify the patterns, and finally predict the possible results. Integration of AI with sample preparation techniques may simplify the decision-making process for practical application.

## Author contributions

All the authors contributed to preparing the manuscript.

## Conflicts of interest

There are no conflicts to declare.

## Supplementary Material
